# Oral delivery of dextran-modified albumin nanoparticles loaded with shikonin for targeted therapy of colorectal cancer

**DOI:** 10.1186/s11671-025-04393-6

**Published:** 2025-11-22

**Authors:** Zhen Ren, Jingyuan Zhao, Shuai Li, Yuan Hong

**Affiliations:** 1 Central Hospital of Dalian University of Technology, Dalian, Liaoning China; 2 Faculty of Medicine, Dalian University of Technology, Dalian, Liaoning China; 3 The First Affiliated Hospital of Dalian Medical University, Dalian, China; 4 Clinical Laboratory Center, Central Hospital of Dalian University of Technology, Dalian, China

**Keywords:** Colorectal cancer, Shikonin, Nanodrugs, Tumor cell metabolism, Targeted delivery

## Abstract

**Background:**

Colorectal cancer (CRC) remains one of the leading causes of cancer-related morbidity and mortality worldwide.

**Methods:**

In this study, we developed a novel nanomedicine-based therapeutic approach targeting key molecules involved in the progression of CRC. By utilizing bioinformatics tools and computer simulations, we identified SLC2A1 and PKM2 as potential therapeutic targets for CRC. Through molecular docking, we confirmed that shikonin (SHK), a bioactive compound derived from traditional herbal medicine, could effectively bind to SLC2A1 and PKM2, indicating its potential therapeutic effect. The novel SHK-loaded nanoplatform, functionalized with albumin (BSA) and glycoside modification (gBSA/SHK), was designed to enhance stability and targeted delivery to tumor sites.

**Results:**

In vitro and in vivo experiments showed that SHK-loaded nanoparticles exhibited good tumoricidal effects on CT26 colorectal cancer cells and shifted tumor cell metabolism.

**Conclusions:**

Overall, our results suggest that SHK-loaded nanodrugs can effectively target key molecular pathways in CRC and provide a promising strategy for colorectal cancer treatment with advantages such as improved drug stability, tumor-specific targeting, and reduced systemic toxicity.

**Supplementary Information:**

The online version contains supplementary material available at 10.1186/s11671-025-04393-6.

## Introduction

Colorectal cancer (CRC) is a malignancy that originates from the colon or rectum and remains a major health concern worldwide. According to data, CRC ranks third among the most prevalent cancers in both males and females, and stands as the second leading cause of cancer-related deaths for both genders [1]. Metabolomics research shows that to meet its demands for growth and proliferation, colorectal cancer cells undergo significant metabolic reprogramming, exhibiting high glucose uptake and unique metabolic characteristics. The Warburg effect is a hallmark of CRC metabolism, which leads to increased glycolysis and lactate production, thereby acidifying the tumor microenvironment. Microorganisms in the microenvironment further promote acidification [2], fostering immune evasion [3], and influences both tumor angiogenesis [4] and cellular metabolism [5].

Shikonin, a naturally active compound derived from the roots of the Lithospermum plant, has been extensively studied. Extensive research confirms that Shikonin acts as a canonical PKM2 pathway inhibitor, effectively blocking the glycolytic pathway in tumor cells, thus inhibiting their growth and proliferation [6]. Furthermore, Shikonin has been shown to regulate tumor cell apoptosis and angiogenesis through multiple mechanisms, including the inhibition of key signaling pathways [7]. Due to its unique anti-tumor properties, Shikonin has garnered significant attention in the scientific community. However, its application as a standalone therapeutic strategy for CRC is restricted by challenges such as limited water solubility, suboptimal bioavailability, potential drug losses before reaching the colon, and insufficient uptake by CRC cells [8]. Albumin, the predominant protein in plasma, is widely used as a drug delivery vehicle because of its inherent biocompatibility, prolonged half-life, and ability to bind various drugs [9]. Notably, CRC cells express specific albumin-related receptors, including GP60, which facilitate enhanced cellular uptake of albumin-drug conjugates. For example, the albumin-paclitaxel conjugate, primarily used for treating breast cancer, non-small cell lung cancer, and pancreatic cancer [10], has shown therapeutic potential in clinical trials for CRC, suggesting a promising direction for CRC therapy [11]. Consequently, albumin-mediated delivery of shikonin has the potential to enhance therapeutic efficacy and reduce side effects.

**Fig. 1:**
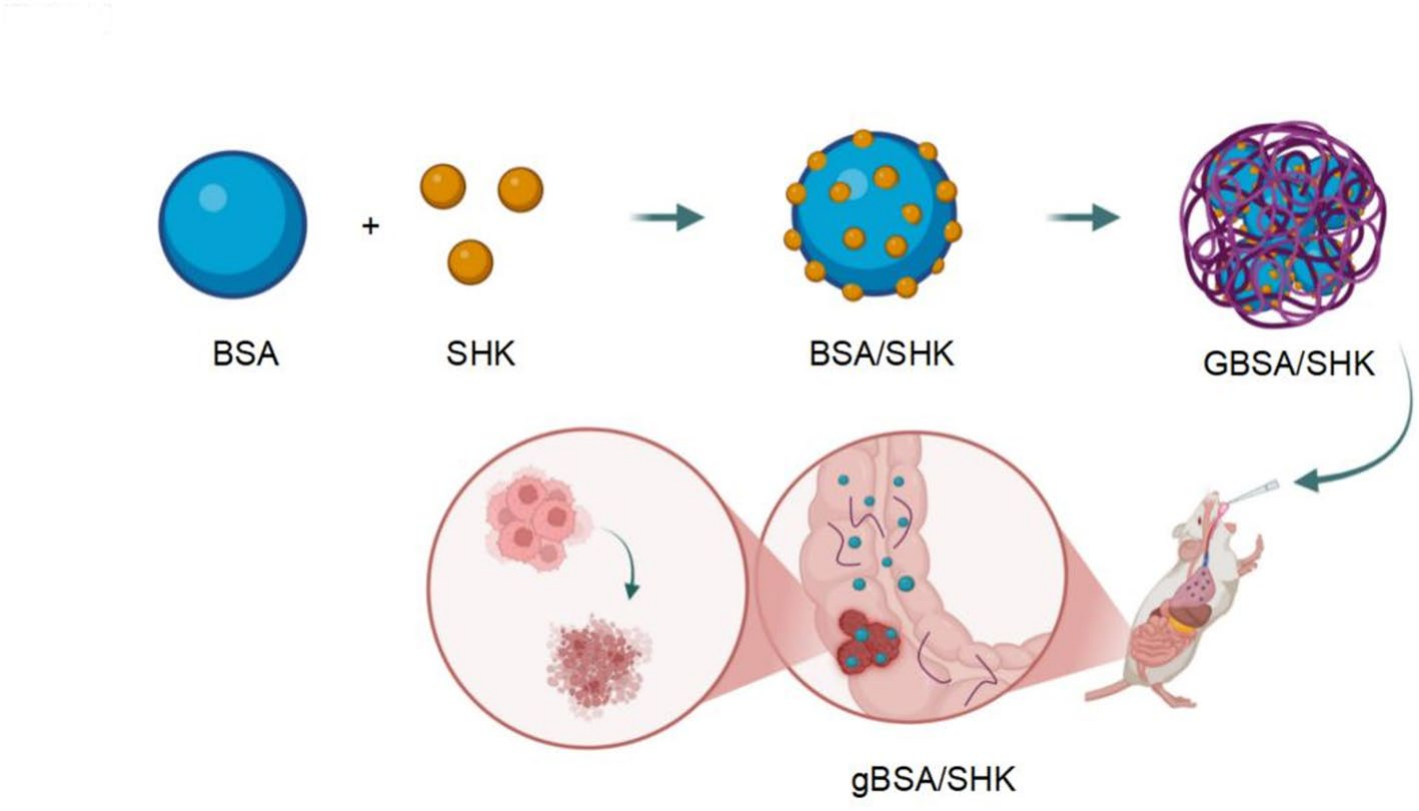
Schematic diagram of targeted drugs. Schematic representation of the targeted drug formulation. BSA and SHK are conjugated to form the BSA/SHK (drug-loaded nanoparticles without dextran loading) complex, which is then coated with glycoside to create the GBSA/SHK (dextran-loaded nanoparticles without enzymatic digestion) formulation. Upon oral administration in mice, the drug reaches the colon, where the glycoside is hydrolyzed, converting the material into gBSA/SHK (dextran-loaded nanoparticles after enzymatic digestion). This formulation is subsequently absorbed by the tumor, exerting its tumor-inhibitory effects

In this study, to enhance the bioavailability of shikonin and take advantage of tumor cells’ high sugar uptake, we loaded shikonin into albumin nanoparticles coated with dextran. The dextran in these nanoparticles is hydrolyzed in the intestine to form glucose-conjugated nanoparticles, which are specifically taken up by tumor cells that have a high demand for sugar. This leads to the release of shikonin within the tumor cells, effectively blocking the Warburg effect in colorectal cancer (CRC), killing the tumor, and significantly improving targeting and safety, potentially leading to better treatment outcomes for tumors (Fig. 1). This research offers a new approach for CRC treatment, which could result in more effective and targeted therapies in the future.

## Materials and methods

### Data sources and data processing

We retrieved colorectal adenocarcinoma patient transcriptome data from The Cancer Genome Atlas (TCGA) database to identify CRC-related targets. Using the R package “limma,” we performed batch correction and screened for differentially expressed genes (DEGs) with |log2(fold change)|> 1 and P value < 0.05.

### Characterizations of nanoparticles

The structure, size, and morphology of nanoparticles were characterized through transmission electron microscopy (TEM) (Thermo Fisher Scientific, Waltham, MA, USA). The size and zeta potential of nanoparticles were measured by a Zeta Sizer Nanoparticle Analyzer (Nano-ZS90, Malvern Panalytical, Malvern, UK). Encapsulation efficiency (EE)% was calculated as EE% = (SHK/SHK initially added to NPs) × 100%.

### Molecular docking

To further understand the drugability of the candidate drug SHK against SLC2A1 and PKM2, we used molecular docking to evaluate the binding energy and interaction pattern between the drug and the target. The lower the docking binding energy, the better the binding effect and affinity, so we will prioritize the drug targets with high affinity for further experimental verification. In this study, we used the protein–ligand docking software AutodockVina1.2.2 (http://autodock.scripps.edu/) for molecular docking. The protein data structure was from the Protein Data Bank (PDB) (http://www.rcsb.org/), and the drug data was from the PubChem compound database (https://pubchem.ncbi.nlm.nih.gov/). All water molecules in the protein and ligand were removed and hydrogen atoms were added. We found the active center of the protein and the docking box size was set to 30 × 30 × 30 nm^3^. Pymol was used for visualization (https://pymol.org/).

### Cell lines

Murine colorectal cancer cells (CT26) were obtained from the Shanghai Cell Bank, Chinese Academy of Sciences (Shanghai, China). CT26 cells were cultured in Dulbecco’s Modified Eagle Medium (DMEM) supplemented with 100 U/L penicillin/streptomycin and 10% fetal bovine serum (FBS), and maintained at 37 °C in a 5% CO2 humidified environment.

### Cell uptake experiment

CT26 cells in the logarithmic growth phase were harvested and seeded in 6-cm culture dishes, allowed to attach for 24 h. Fluorescein isothiocyanate (FITC)-labeled BSA-NP and BSA-Glu were introduced, and cells were collected after 4 h of incubation. After PBS washing, intracellular fluorescence intensity was quantified using flow cytometry. CT26 cells in the logarithmic growth phase were harvested and seeded into 24-well culture plates with cell climbing slides. After 24 h, fluorescein isothiocyanate (FITC)-labeled BSA/SHK and gBSA/SHK were applied and incubated for 4 h before cell collection. After PBS washing, cells were fixed with 4% paraformaldehyde, permeabilized with 0.25% Triton X-100, and stained with Hoechst 33342 (C1025, Beyotime, China) for nuclear staining and Actin-Tracker Red-594 (C2205S, Beyotime, China) for cytoskeleton staining. Following PBS washing, the slides were sealed, and fluorescence intensity within the cells was examined under a fluorescence microscope.

### Cell proliferation assays

CT-26 cells in the logarithmic growth phase were counted and seeded at a density of 10,000 cells per well in a 96-well plate with 100 μl of culture medium. After 24 h of incubation, cells were treated with specified drug concentrations and further incubated for 48 h. Cell proliferation was evaluated using the Cell Counting Kit-8(GK10001, GLPBIO, China).

### Colony formation

Cells in the logarithmic growth phase were seeded at a density of 1,000 cells per well in a 12-well plate. After 2 days of incubation, cells were treated with designated drug concentrations. Following 14 days of incubation, colonies were washed with phosphate-buffered saline (PBS), fixed with anhydrous ethanol, and stained with crystal violet. Colony counting was performed, with each group replicated in triplicate.

### Cell viability assays

CT26 cells in the logarithmic growth phase were seeded in 96-well plates and cultured for 24 h. Subsequently, cells were treated with specified drug concentrations for an additional 24 h. Cell viability was assessed using the Calcein/PI Cell Viability/Cytotoxicity Assay Kit (C2015S, Beyotime, China).

### Lactate assays

CT26 cells in the logarithmic growth phase were harvested and seeded into 6-well culture plates for 24 h, then treated with appropriate drug concentrations for an additional 24 h. After collecting the cells, washing with PBS, and processing the samples with the CheKineTM Micro Lactate Assay Kit (KTB1100, Abbkine, China), the OD value at 450 nm was measured to analyze lactate metabolism levels.

### Nuclear and cytoplasmic protein extraction and Western blot

The nuclear and cytoplasmic proteins were obtained using the Nuclear and Cytoplasmic Protein Extraction Kit (P0028, Beyotime, China) following the manufacturer’s instructions. Western blotting was performed as described previously. Briefly, equal amounts of extracts were loaded onto the SDS polyacrylamide gels, electrophoresed, and blotted onto the PVDF membranes (IPVH00010, Millipore, Germany). The membrane was blocked with 5% skimmed milk, followed by incubation with primary antibodies at 4 °C overnight. Then, the membranes were incubated with the HRP-conjugated secondary antibodies and detected using an ECL kit (P0018FM, Beyotime, China). The primary antibodies included anti-c-Myc (CQA8120, Cohesion, China), anti-Cyclin D1 (CQA6577, Cohesion, China) and beta Actin Rabbit Polyclonal Antibody (Catalog# R1207-1, HUABIO, China). The second antibodies included goat anti-rabbit (CSA2115, Cohesion, China) and anti-Mouse (CSA2107, Cohesion, China).

### Mitochondrial membrane potential measurement

Use the enhanced mitochondrial membrane potential measurement kit (Cat#C2003S, Beyotime, China). Add 1 mL of JC-1 staining buffer to dilute JC-1 for every 5 µL of JC-1 (200 ×). Inoculate cells at a density of 2 × 10^5^ cells/well after different drug treatments in confocal dishes. After drug treatment, remove the dish and wash with PBS. Then add 1 mL of cell culture medium and 1 mL of JC-1 staining solution, incubate at 37 ℃ for 20 min, then remove the supernatant and wash the cells with JC-1 staining buffer. Finally, add 2 mL of cell culture medium and observe under a laser confocal microscope (LSM980, ZEISS, Germany). Set the excitation wavelength to 490 nm and the emission wavelength to 530 nm to detect JC-1 monomers. Set the excitation wavelength to 525 nm and the emission wavelength to 590 nm to detect JC-1 aggregates.

### Detection of cellular ROS by DCFH/DCFH-DA

Intracellular ROS generation was measured by DCFH-DA (C2938, Throme, USA). DCFH-DA can diffuse rapidly across the cell membrane and is deacetylated by cellular esterases to non-fluorescent DCFH, which is then rapidly oxidized to fluorescent 2′,7′-dichlorofluorescein (DCF) in the presence of ROS. Cells were seeded at a density of 2 × 10^5^ cells/well after different drug treatments in confocal microplates. After drug treatment, cells were washed with 100 µL/well PBS and incubated with 200 µL 10 µm DCFH-DA in Hank’s balanced salt solution (HBSS) containing 1% FBS for 30 min. Finally, cells were rinsed and stained with Hoechst 33342 for 0.5 h. After washing twice with PBS, the fluorescence images of cells with different treatments were observed using a laser confocal microscope (LSM980, ZEISS, Germany).

### Lyso-tracker red staining

The Lyso-Tracker Red staining (Cat. No. C1046, Beyotime, China) was performed to measure the presence of lysosomes, according to the supplier’s instructions. The cells were incubated with Lyso-Tracker Red (50 nM) at 37 °C for 30 min. Then, the images were captured using confocal microscopy (LSM980, ZEISS, Germany).

### Pyruvate acid (PA) assays

CT26 cells in the logarithmic growth phase were harvested and seeded into 6-cm culture plates, then treated with appropriate drug concentrations for 24 h. Cells were collected, washed with PBS, and assessed using the CheKineTM Micro Pyruvate Acid (PA) Assay Kit (KTB1121, Abbkine, China). PA content was analyzed by measuring the OD value at 520 nm.

### Transcriptomic analysis

RNA sequencing was performed on NextSeq500 (Illumina, USA) as previously described. RNA-seq data were processed using TopHat2 and Cufflinks v2.1.1. Gene isoforms were summed up to obtain fragments per kilobase of exon model per million reads mapped (FPKM) values for each gene and only protein-coding genes, as defined by the HUGO Gene Nomenclature Committee, were retained. Log2-fold changes (LFC) in gene expression between autologous and allogenic TILs in the above co-culturing experiments were calculated by subtracting the gene values between these experimental conditions after standard normalization and log-transformation. Analysis was not adjusted for tumor type or clinical characteristics.

### Gene ontology (GO) and Kyoto encyclopedia of genes and genomes (KEGG) enrichment analysis

To explore potential mechanisms related to CRC, we performed Gene Ontology (GO) and Kyoto Encyclopedia of Genes and Genomes (KEGG) enrichment analyses using DAVID (https://david.ncifcrf.gov/).

### In vivo study

Male BALB/c nude mice (6 weeks old) were purchased from Liaoning Changsheng Biotechnology Co., Ltd. (Liaoning, China). All animal experiments were approved by the Experimental Animal Ethics Committee of Dalian Medical University (No. AEE22108; November 6, 2023). 5 × 10^6^ CT26 cells suspended in 100 μl PBS and mixed with Matrigel (356234, Corning, USA) were orthotopically inoculated into the rectum of mice. Seven days after inoculation, mice were randomly divided into groups (6/group). Shikonin and nanomaterials were gavaged once every 3 days in a volume of 100 μl. The size of tumors in mice was observed by in vivo imaging every 9 days. All mice were killed after 18 days of the experiment. Blood was collected for biomedical measurements. Tumors and their major organs were collected and fixed with 4% paraformaldehyde.

### H&E staining and immunohistochemistry

Lung tissues containing tumor nodules were embedded in 4% methanol-free formaldehyde for 48 h. Subsequently, the tumor nodules in lung tissues were assessed in 4 μm-thick hematoxylin and eosin (H&E)-stained sections. To evaluate tumor angiogenesis and proliferation, the expression of Ki67 was assessed by staining with anti-Ki67 mAb (HA721115, HUABIO, China). Images of H&E staining and immunohistochemistry were obtained under a light microscope.

### Statistical analysis

All analyses were performed using GraphPad Prism 7.0 software (CA, USA). Data were presented as mean ± standard error of the mean (SEM) unless otherwise stated. The two-tailed t test was used to analyze the independent samples between groups. Statistical significance was defined as *P < 0.05, **P < 0.01, and ***P < 0.001.

## Results

### Target analysis and material characterization

**Fig. 2:**
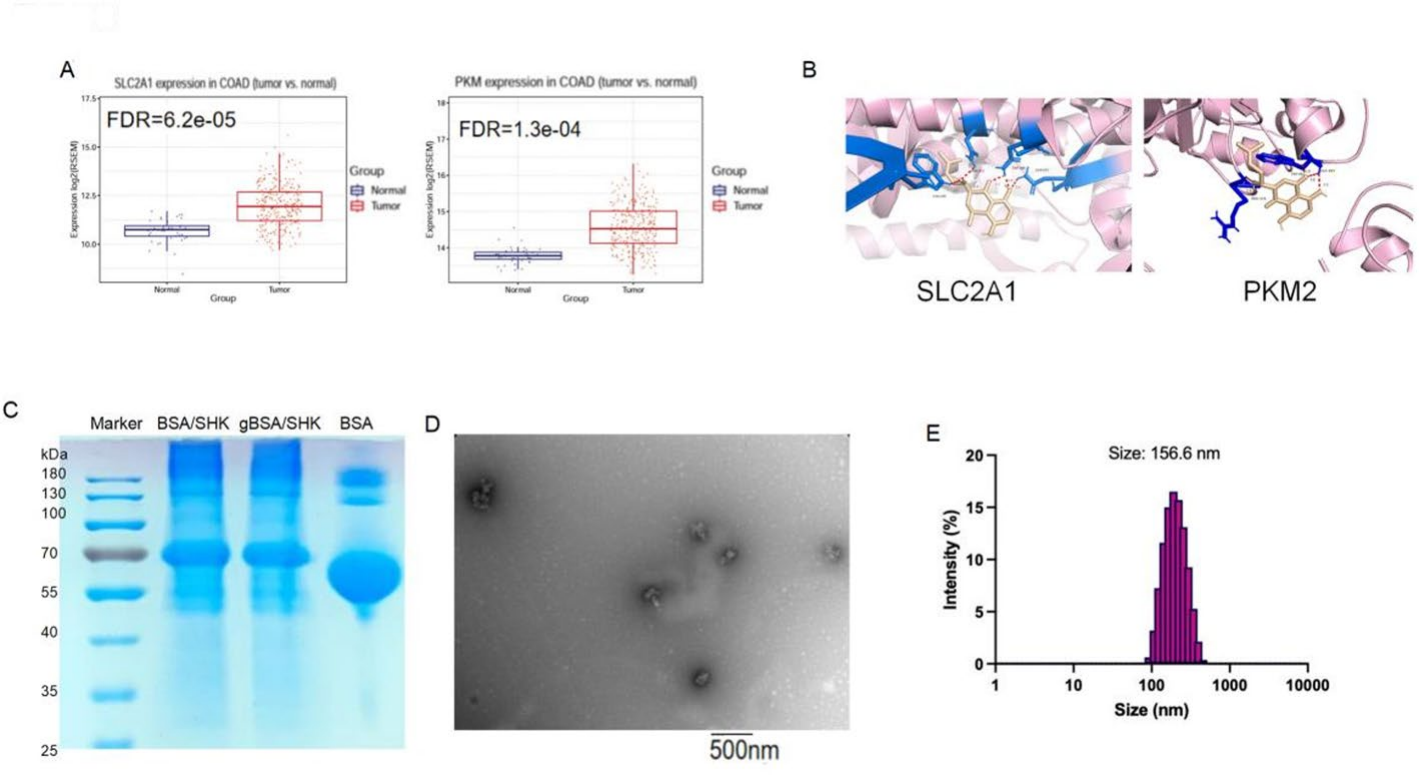
Target analysis and material characterization. **A** Expression differences of two genes between tumor patients and normal individuals. **B** 3D schematic of the docking of two genes with SHK drug molecules. **C** Electrophoresis analysis of nanomaterial consistency. **D** Representative TEM (Transmission Electron Micrograph) images of GBSA/SHK. **E** Nanoparticle size distribution

The researchers learned from the literature that SLC2A1 (GLUT1) and PKM2, as two popular SHK binding sites, have not been studied in colorectal cancer [12]. Therefore, to further confirm the differential expression of these two genes in CRC, we performed additional analyses using an online tool (https://guolab.wchscu.cn/GSCA/#/) and verified the differential expression of SLC2A1 and PKM2 in tumors and normal tissues in the TCGA database (Fig. 2A). Subsequently, molecular docking simulations were performed to evaluate the binding affinity of SHK to these targets. The docking scores of both genes with SHK were less than -5, indicating significant binding. The three-dimensional molecular model showed that both SLC2A1 and PKM2 formed more than four hydrogen bonds with a binding energy less than -2.0 kcal/mol, further supporting the practical relevance of these interactions (Table 1 and Fig. 2B). Based on these analyses, PKM2 and SLC2A1 were confirmed as CRC therapeutic targets, and SHK was selected for experimental treatment. After the material was synthesized, the size of the polypeptide molecule was first verified by electrophoresis. The results showed that the size of the undigested BSA/SHK and gBSA/SHK were about 70 KDa (Fig. 2C), which was consistent with expectations. Subsequent transmission electron microscopy revealed GBSA/SHK of approximately 150 nm in size (Fig. 2D), consistent with the average particle size of 156.6 nm obtained by particle size analysis (Fig. 2E). The drug loading efficiency of BSA/SHK (drug-loaded nanoparticles without dextran loading) was determined to be 3.1%. After 24 h of treatment in simulated intestinal fluid, the particle size of gBSA/SHK was reduced (Supplementary Fig. 1A).

**Table 1:** Target gene molecular docking score

Target	SMILES	Compounds	Docking Score
SLC2A1	CC(=CC[C@H](C1=CC(=O)C2=C(C=CC(=C2C1=O)O)O)O)C	Shikonin	-9.2
PKM2	CC(=CC[C@H](C1=CC(=O)C2=C(C=CC(=C2C1=O)O)O)O)C	Shikonin	-7.7

### gBSA/SHK is internalized into tumor cells through endocytosis

**Fig. 3:**
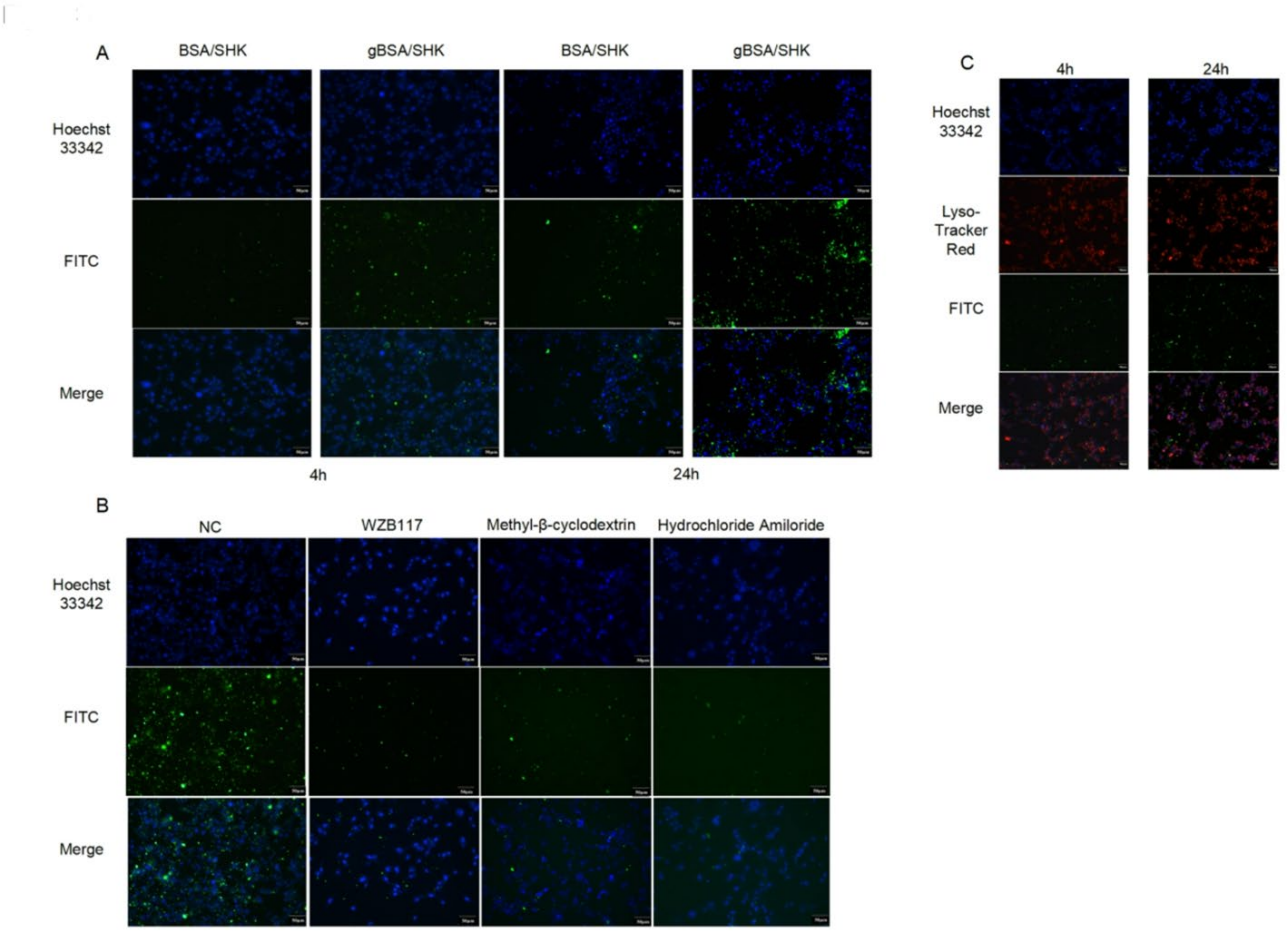
gBSA/SHK is Internalized into Tumor cells Through Endocytosis. **A** Fluorescence staining at different co-incubation times (4 h, 24 h) was observed using laser confocal microscopy. Hoechst33342 was used for cell nuclei, and FITC for BSA/SHK and gBSA/SHK. **B** Fluorescence staining of cells after co-incubation with various inhibitors (WZB117, Methyl-β-cyclodextrin, and Hydrochloride Amiloride) observed by laser confocal microscopy. Hoechst33342 was used for cell nuclei, and FITC for gBSA/SHK. **C** Fluorescence staining of lysosomes after co-incubation for different times (4 h, 24 h). Hoechst33342 for cell nuclei, Lyso-Tracker Red for lysosomes, and FITC for gBSA/SHK

This study investigates whether nanomaterials can enter tumor cells via endocytosis by evaluating the uptake of BSA/SHK and gBSA/SHK by colon cancer cells. As anticipated in the in vivo environment, glycan chains are digested by intestinal fluid, leading to predominant endocytosis of gBSA/SHK in tumor cells. Uptake was directly observed using confocal microscopy, where blue fluorescence represents Hoechst stained nuclei, and green fluorescence indicates FITC-labeled nanoparticles. Results show that differences in endocytosis of BSA/SHK and gBSA/SHK by tumor cells begin to manifest from the fourth hour, peak at the 24-h mark (Fig. 3A). In addition, the flow cytometry results of CT26 also confirmed this (Supplement Fig. 1B). Furthermore, addition of the glucose transporter 1 (GLUT1) inhibitor WZB117 to the culture medium significantly reduced the mean fluorescence level in CT26 cells, indicating that the decreased glucose uptake capacity of the cells also inhibited the uptake of gBSA/SHK by colon tumor cells (Fig. 3B). To eliminate confounding factors such as adhesion, endocytosis inhibitors (methyl-β-cyclodextrin and amiloride hydrochloride) were also added to the culture medium. After 24 h of co-incubation of CT26 cells with gBSA/SHK, confocal microscopy revealed a significant decrease in the mean fluorescence intensity in CT26 cells compared to the control group, indicating that gBSA/SHK uptake by colon tumor cells was inhibited (Fig. 3B). In addition, staining of lysosomes showed that the nanomaterials could enter the lysosomes and exert their effects as planned (Fig. 3C). Therefore, the uptake of gBSA/SHK by CT26 cells was mainly mediated by endocytosis, which simulated the process of glucose uptake pathway, which was consistent with expectations.

### Tumor cell killing efficacy based on gBAS/SHK

**Fig. 4:**
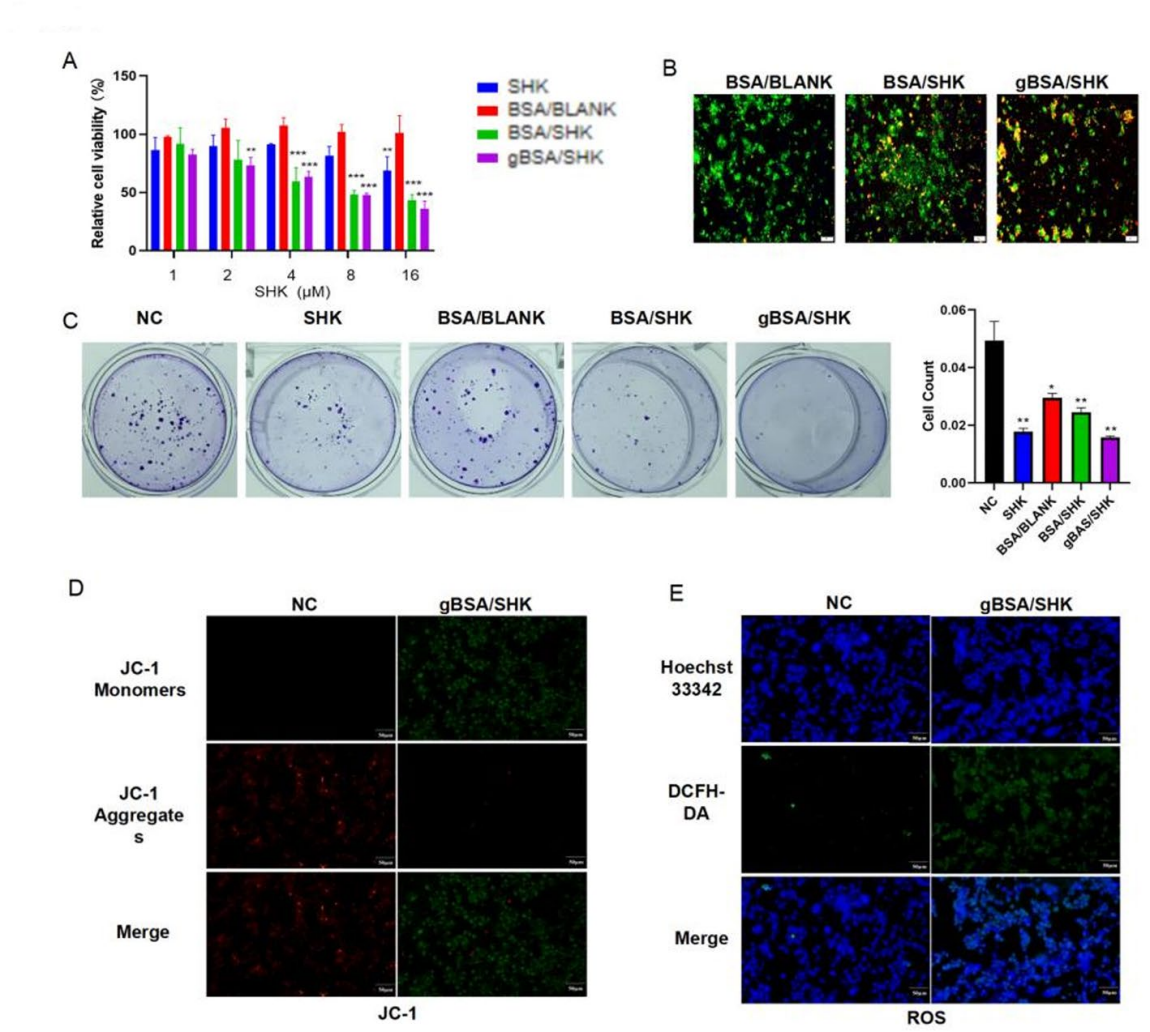
Tumor Cell Killing Efficacy Based on gBAS/SHK.** A** Cytotoxicity of different drugs (SHK, BSA/BLANK, BSA/SHK, and gBSA/SHK) was evaluated using the MTT assay at concentrations of 1, 2, 4, 8, and 16 μM on CT26 cells. **B** Fluorescence microscopy images showing cell killing effects of drugs with different contents. **C** CT26 cells were co-incubated with different drugs (SHK, BSA/BLANK, BSA/SHK, and gBSA/SHK) for plate cloning and their statistical graphs. **D** JC-1 staining was performed to assess changes in mitochondrial membrane potential using fluorescence microscopy. The excitation wavelength was set to 490 nm, and the emission wavelength to 530 nm to detect JC-1 monomers; for JC-1 aggregates, the excitation wavelength was 525 nm, and the emission wavelength was 590 nm. **E** Intracellular ROS generation was measured using DCFH-DA, and fluorescence images were captured using laser confocal microscopy. Hoechst33342 was used for staining cell nuclei. *P < 0.05, **P < 0.01, ***P < 0.001

The MTT method was used to detect the cytotoxicity of SHK, BSA/BLANK, BSA/SHK(Nanocomplex without added dextran), and gBSA/SHK at different concentrations on CT26 colon cancer cells. When the concentration of BSA/BLANK exceeded 16 µM and was co-cultured with cells for 48 h, the survival rate of CT26 cells remained above 95%, indicating that drug-free nanoparticles were safe (Fig. 4A). At the same time, at the same drug concentration, the cell survival rate of the SHK group was always higher than that of the BSA/SHK and gBSA/SHK groups. This was due to the uptake of nanomaterials by CT26 cells, which led to increased accumulation of SHK in cells (Fig. 4A). After administration, the effect of nanomaterials on CT26 cells was observed using a fluorescence microscope, and the results showed the green fluorescent gBSA/SHK was mainly located in the cell membrane stained red. This indicates that gBSA/SHK is more likely to act on CT26 in the same amount of time after glycan hydrolysis (Fig. 4B). The colony formation assay was used to evaluate the cell proliferation capacity under drug treatment conditions. After treatment, a significant decrease in the number of colonies was observed, indicating that the drug was effective in preventing the proliferation of CT26 cells (Fig. 4C). After the experiment proved that gBSA/SHK can effectively kill cancer cells, we further explored its potential mechanism. SHK can induce apoptosis by generating ROS, disrupting membrane potential, and inducing mitochondrial dysfunction [13]. Therefore, the mitochondrial membrane potential and ROS concentration of CT26 cells after drug treatment were evaluated. After treatment, mitochondria stained with JC-1 showed green fluorescence, indicating a decrease in mitochondrial membrane potential (Fig. 4D). Similarly, ROS staining increased significantly after drug treatment, indicating a high intracellular ROS concentration (Fig. 4E). Consistent with the reported results, this indicates that SHK in the nanomaterial has been successfully absorbed and active in the cell.

### Metabolic and transcriptome analysis of CT26 cells post drug treatment

**Fig. 5:**
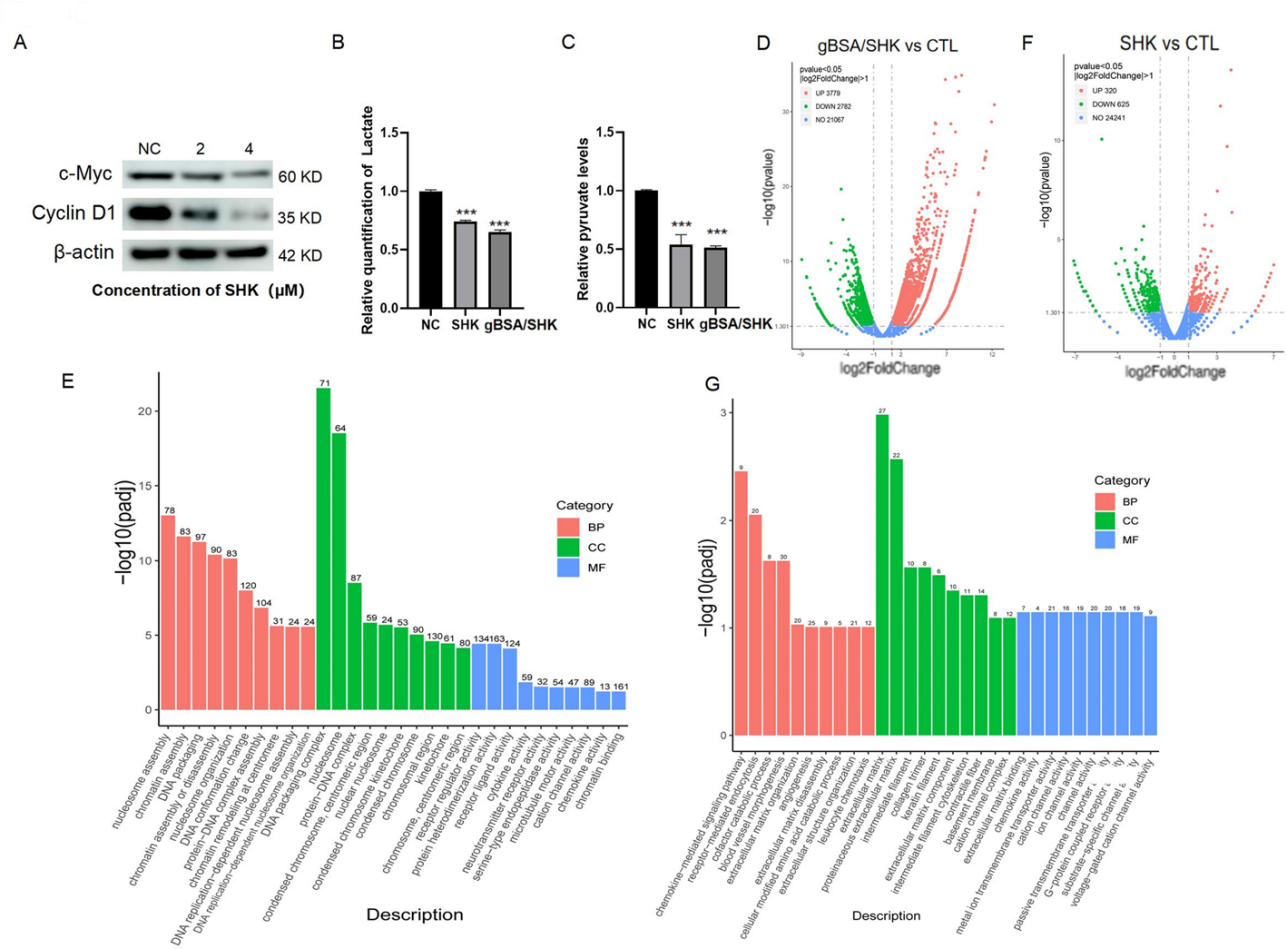
Metabolic and transcriptome Analysis of CT26 Cells Post Drug Treatment. **A** Western Blot analysis of intracellular c-Myc and Cyclin D1 proteins in CT26 cells after 24 h of treatment with different concentrations of gBSA/SHK. **B**, **C** Metabolomics analysis of lactate and pyruvate and lactate levels in CT26 cells treated with different drugs (SHK, gBSA/SHK (digested drug-loaded nanoparticles). **D**, **E** Gene ontology (GO) analysis of CT26 cells co-incubated with gBSA/SHK and compared with the control group. **F**, **G** Gene Ontology (GO) analysis of CT26 cells co-incubated with gBSA/BLANK compared with the control group. *P < 0.05, **P < 0.01, ***P < 0.001

PKM2 is a downstream molecule of hypoxia-inducible factor 1α (HIF-1α) and a key enzyme involved in aerobic glycolysis of cancer cells. Inhibition of PKM2 can inhibit cancer cell proliferation by reducing intracellular ATP levels [14]. To investigate whether the nanomaterials inhibited tumor cell glycolysis as expected, we first performed Western blotting to verify the expression of PKM2 downstream target proteins, c-Myc and Cyclin D1. The results showed a significant decrease in downstream protein expression with increasing drug concentration (Fig. 5A). This preliminary evidence supports the tumor-killing mechanism of gBSA/SHK. Simultaneously, the pyruvate concentration in CT26 cells was measured after drug treatment. Compared with the control group, both gBSA/SHK and SHK treatment groups showed lower lactate and pyruvate levels (Fig. 5B, C), indicating that SHK effectively blocked the aerobic glycolysis pathway. The trend of regulating these biomolecules was consistent among different treatment groups, indicating that the mechanism by which these SHK-loaded nanoparticles kill tumor cells is by disrupting aerobic glycolysis. Following preliminary investigations into the mechanism by which gBSA/SHK kills cancer cells, we conducted whole-genome sequencing of the treated cells to understand specific alterations in gene expression within the tumor cells. Compared to the control group, the non-SHK group exhibited significant increases in both upregulated and downregulated pathways. Gene Ontology (GO) analysis revealed notable the SHK group showed active GO processes related to extracellular matrix degradation, cell modification, amino acid decomposition metabolism, and inhibition of extracellular matrix and protein interactions (Fig. 5D, E), correlating with SHK-induced apoptosis and inhibition of aerobic glycolysis. Conversely, enrichment in pathways related to chromatin protein, nucleosome assembly, and DNA packaging, all implicated in cellular replication processes (Fig. 5F, G). This suggests more robust cellular activity in the non-SHK group, potentially due to the absence of cytotoxicity and the presence of BSA, which tumor cells can utilize as an energy source. The differential gene expression count also reflected the same trend (Supplement Fig. 1C). Subsequent KEGG analysis indicated that in the non-SHK group, activities of various channels and receptors, including voltage-gated cation channels, substrate-specific channels, G-protein-coupled receptors, and passive transmembrane transporters, were enhanced, along with active receptor-mediated endocytosis, all indicating vigorous cellular activity related to the internalization of BSA (Supplement Fig. 1D). However, regulatory effects in the SHK group were predominantly associated with chromosomal, centromeric, nucleosomal, and other nuclear components related to DNA replication (Supplement Fig. 1E), consistent with reports in the literature regarding the direct cellular action of PKM2 [15]. The aforementioned experiments collectively indicate that gBSA/SHK can be internalized by cells and exert cytotoxic effects through the action of SHK.

### Inhibition of xenograft tumors by NPs therapy in vivo and its biosafety

**Fig. 6:**
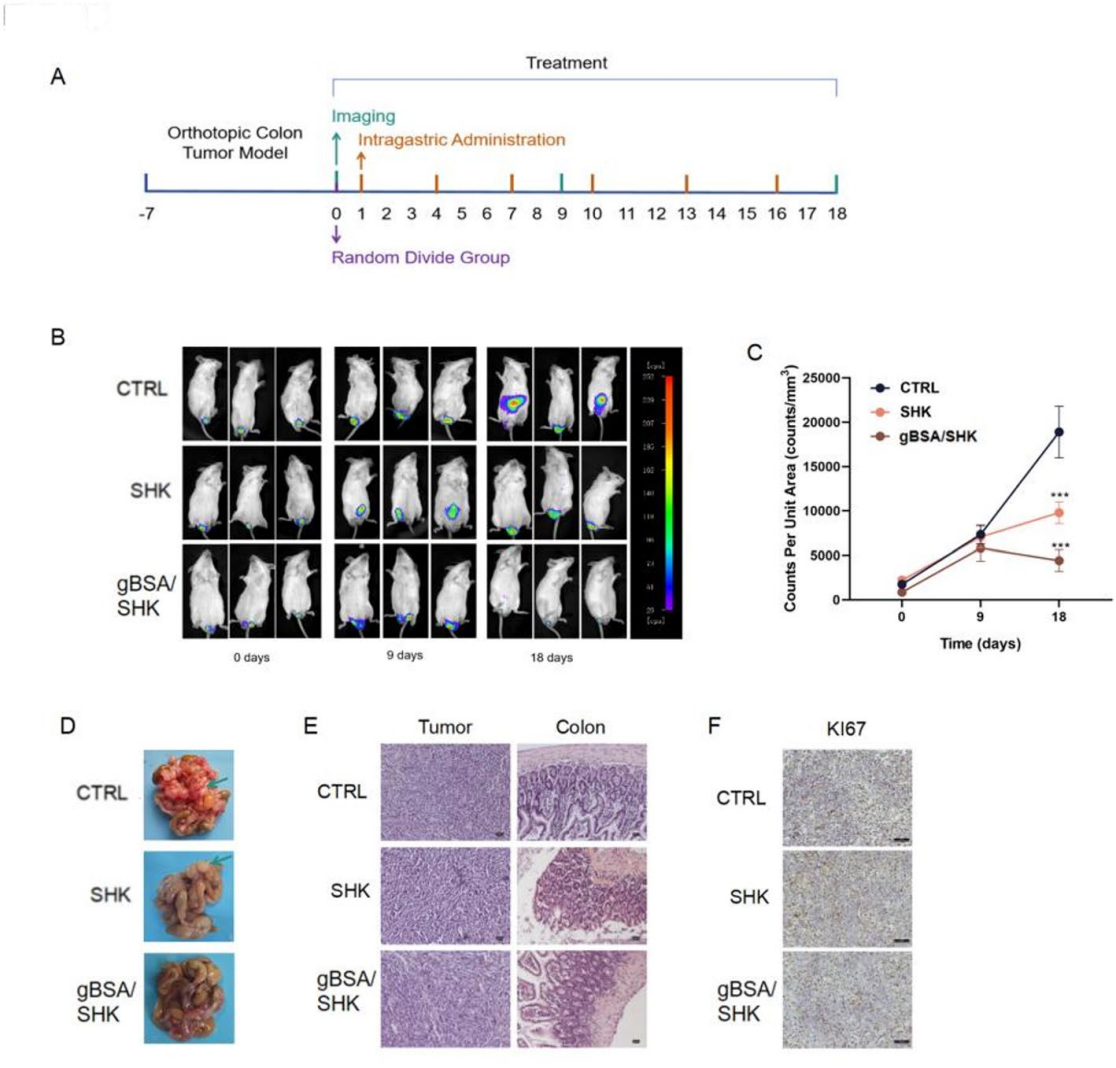
Inhibition of xenograft tumors by NPs therapy in vivo and its biosafety. **A** In vivo experimental schedule (tumor inoculation 7 days prior to the experiment, grouping on day 0, imaging on days 0, 9, and 18, drug administration on days 1, 4, 7, 10, 13, and 16). **B** In vivo imaging results of CT26 tumor-bearing mice at different times (0, 9, and 18 days) following treatment with different drugs (CTRL, SHK, gSHK/BSA). **C** Brightness analysis per unit area of in vivo imaging at days 0, 9, and 18 in CT26 tumor-bearing mice treated with different drugs (CTRL, SHK, gSHK/BSA) by oral gavage. **D** Intestinal dissection of CT26 tumor-bearing mice at day 18 post-treatment with different drugs (CTRL, SHK, gSHK/BSA) (arrow indicates a prominent tumor). **E** H&E staining analysis of tumor sites and colons from mice treated with different drugs (CTRL, SHK, gSHK/BSA). **F** KI67 staining analysis of tumor sites from mice treated with different drugs (CTRL, SHK, gSHK/BSA). *P < 0.05, **P < 0.01, ***P < 0.001.

The impact of NP treatment on CT26 tumor growth was assessed in an in vivo experiment. Tumor-bearing mice were orally administered equivalent concentrations of SHK + dBET6, CMS, SHK, and gBSA/SHK combinations, or an equal volume of saline (control), every 3 days, and in vivo imaging was conducted on days 0, 9, and 18 (Fig. 6A). Compared with the control group and SHK group, the results of in vivo imaging showed that the gBSA/SHK group effectively inhibited tumor growth (Fig. 6B, C). At the same time, the anatomical results on the 18th day also showed that the orthotopic tumors on the colorectum of mice in the gBSA/SHK group were significantly smaller than those in the control group and SHK group (Fig. 6D). In addition, HE staining (Fig. 6E) and KI67 staining (Fig. 6F) showed that the nanodrug had a significant cytotoxic effect on tumors. HE staining of other organs (heart, liver, lung, kidney and spleen) showed that the nanomaterial exhibited high biosafety (Supplement Fig. 2A). The hemolysis experiment of GBSA/SHK material shows that it exists stably in the blood and does not produce hemolysis (Supplement Fig. 1F). There was no significant difference in blood biochemical results among different groups Supplement Fig. 2B). Finally, the body weight of mice in different drug groups remained relatively stable (Supplement Fig. 2C). This shows that the experimental drug exhibits high biosafety.

## Discussion

Colorectal cancer (CRC), the third most common malignant tumor worldwide, poses a significant threat to human health, characterized by the proliferation of malignant cells in colonic epithelium [16, 17]. Despite significant advancements in treatment strategies, such as immunotherapy [18], the primary treatments for CRC remain surgery and chemotherapy, the latter being crucial for patients with advanced CRC [19]. Currently, chemotherapeutic agents for CRC treatment include 5-fluorouracil (5-FU) [20], capecitabine, oxaliplatin, and irinotecan [21]. However, these chemotherapeutic drugs have significant limitations, including high dosage requirements, severe side effects, and notable drug resistance, which reduce patients’ survival time [22]. Therefore, developing new therapeutic strategies to enhance antitumor efficacy is both necessary and important. In this study, we developed a albumin nanoparticles coated with dextran. Albumin nanoparticles coated with sugar wraps target tumor cells, enhancing shikonin uptake and reducing metabolic activity. This approach alleviates tumor growth and immune suppression from the Warburg effect, ultimately leading to tumor cell death and inhibited tumor growth.

Shikonin (SHK) is a naturally occurring naphthoquinone pigment, and it is the main active compound isolated from the traditional Chinese herb Lithospermum erythrorhizon [23]. It exhibits potential antitumor activity by specifically inhibiting PKM2 in cancer cells, achieving an inhibition rate of over 50% [24, 25]. However, SHK has several drawbacks, including multiple targeting effects, instability, high toxicity, and poor solubility, which hinder its clinical application [26]. To address these issues, scientists have synthesized various shikonin analogs with antitumor activity, including acyl shikonin derivatives with cyclopropyl groups [27], aminoglucoside shikonin glycosides [28], naphthoquinone derivatives (COMP3-24) [29], and benzofuran derivatives (1AA) [30]. However, none of these have demonstrated outstanding clinical efficacy. Therefore, it is necessary to develop new targeted shikonin drugs. This study significantly improved the bioavailability and targeting of SHK, allowing it to directly reach the intestinal tumor site and target and kill tumor cells.

BGNPs take advantage of the specificity of the tumor microenvironment and metabolism to achieve targeted effects on tumor sites, improving safety and effectiveness. Reprogramming of energy metabolism has long been considered a hallmark of cancer [31]. Even in the presence of sufficient oxygen, many cancer cells metabolize glucose through aerobic glycolysis rather than oxidative phosphorylation, a phenomenon known as the “Warburg effect” [32, 33]. As research progresses, scientists have found that the PKM2 gene is highly associated with the Warburg effect in tumors [34]. Upregulation of PKM2 or the replacement of PKM1 with PKM2 triggers aerobic glycolysis, a process observed in various cancers [35]. Unlike PKM1, which retains high constitutive PK activity, PKM2’s low activity allows cancer cells to accumulate various glycolytic intermediates necessary for the biosynthesis of nucleic acids, amino acids, and phospholipids, promoting anabolism through the pentose phosphate pathway [36, 37]. Additionally, accumulated PKM2 in the cytoplasm can translocate to the nucleus, where it acts as a protein kinase and transcriptional coactivator, regulating the transcription of oncogenic and metabolic genes, leading to rapid cell proliferation and tumorigenesis [38–40]. Therefore, we exploit high glucose uptake in colorectal cancer cells to direct targeted drug modification, enabling the drug to enter tumor cells via this effect, induce cytotoxicity, reduce tumor cell metabolism, and ultimately inhibit tumor growth.

## Conclusion

Researchers designed a nanoparticle (gBSA/SHK) loaded with SHK, significantly enhancing cytotoxicity in CT26 colorectal cancer cells by inducing apoptosis, disrupting mitochondrial function, and inhibiting aerobic glycolysis. This promising colorectal cancer treatment strategy was validated through both in vitro and in vivo experiments. This study contributes to the field by providing a novel nanoparticle drug delivery system that significantly enhances the bioavailability and antitumor effects of shikonin, overcoming some limitations of current chemotherapeutic agents. Despite these results, our study has certain limitations. The in vivo experiments were conducted on a limited number of animal models, and further studies are needed to confirm these findings in larger and more diverse populations. Additionally, the long-term stability and potential toxicity of gBSA/SHK nanoparticles in vivo remain to be thoroughly investigated.

## Supplementary Information

Below is the link to the electronic supplementary material.

Supplementary Material 1

## Data Availability

The study utilized publicly available data, which can be openly accessed.

## Abbreviations

BSA: Albumin
CRC: Colorectal Cancer
DCF: Dichlorofluorescein
DMEM: Dulbecco’s modified eagle medium
FBS: Fetal bovine serum
FPKM: Fragments Per Kilobase of exon model per Million mapped fragments
FITC: Fluorescein isothiocyanate
GO: Gene ontology
HBSS: Hank’s balanced salt solution
H&E: Hematoxylin and eosin
KEGG: Kyoto encyclopedia of genes and genomes
LFC: Log_2_-fold changes
PA: Pyruvate acid
PBS: Phosphate-buffered saline
SHK: Shikonin
TCGA: The cancer genome atlas
TEM: Transmission electron microscopy
SEM: Standard error of the mean
