# Supplementary material for: Oral delivery of dextran-modified albumin nanoparticles loaded with shikonin for targeted therapy of colorectal cancer
Source: Discov Nano. 2025 Nov 22;20(1):210. doi: 10.1186/s11671-025-04393-6 (PMC12638471; doi:10.1186/s11671-025-04393-6)
Supplement: Supplementary file 1 — Supplementary Material 1 [file 11671_2025_4393_MOESM1_ESM.docx]

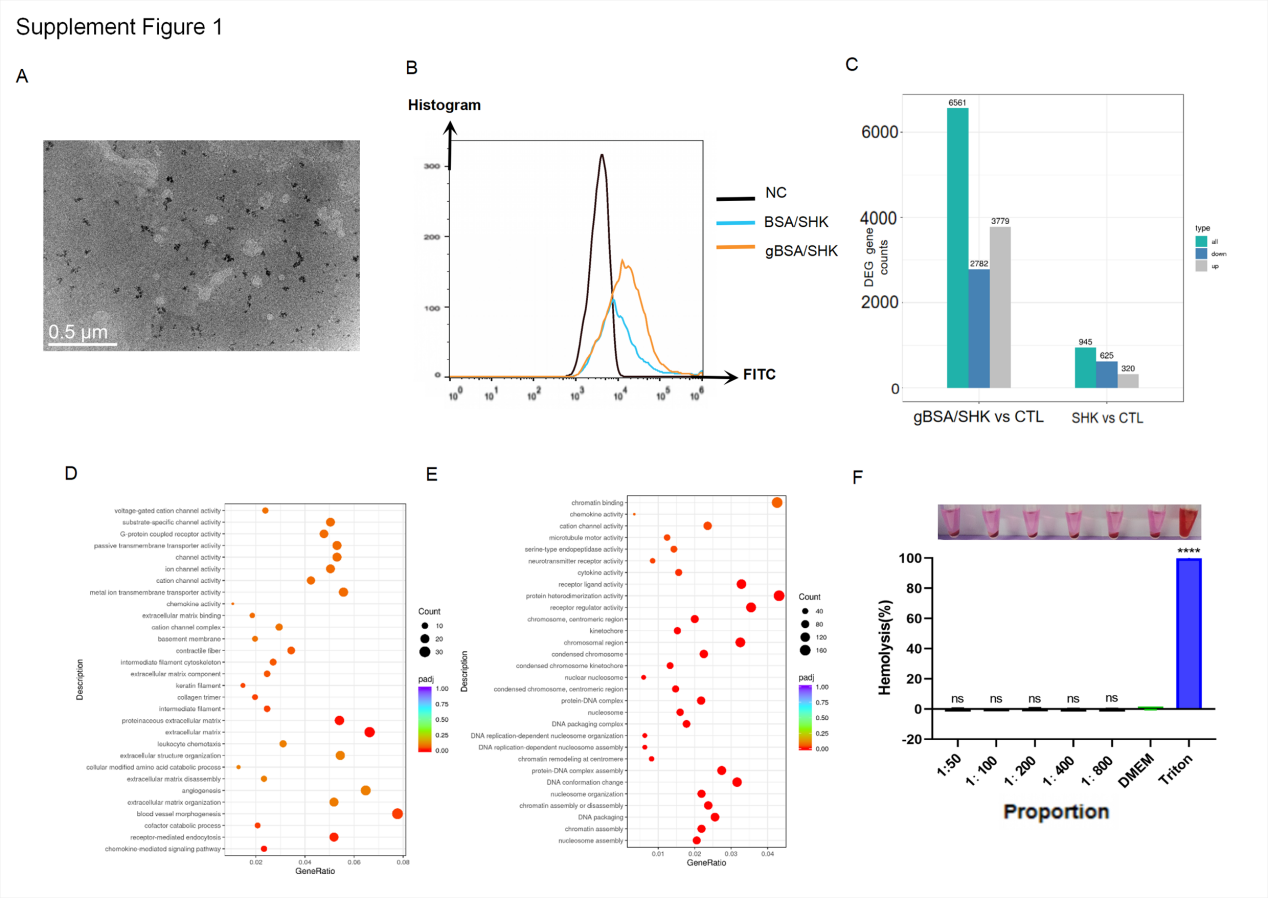


**Supplement Figure 1**

**A** Representative TEM images of gBSA/SHK after 24 hours of treatment with simulated intestinal fluid. **B** Flow cytometry results of CT26 cells with GBSA/SHK and gBSA/SHK with Fitc.  **C** Number of differentially expressed genes in CT26 cells after co-incubation with the two drugs compared with the control group. **D** KEGG analysis of CT26 cells co-incubated with gBSA/BLANK and compared with the control group. **E** KEGG analysis of CT26 cells co-incubated with gBSA/SHK and compared with the control group. **F** Hemolysis test of GBSA/SHK with different ratios and control group (DMEM and Triton).**P* < 0.05, ***P* < 0.01, ****P* < 0.001.

**
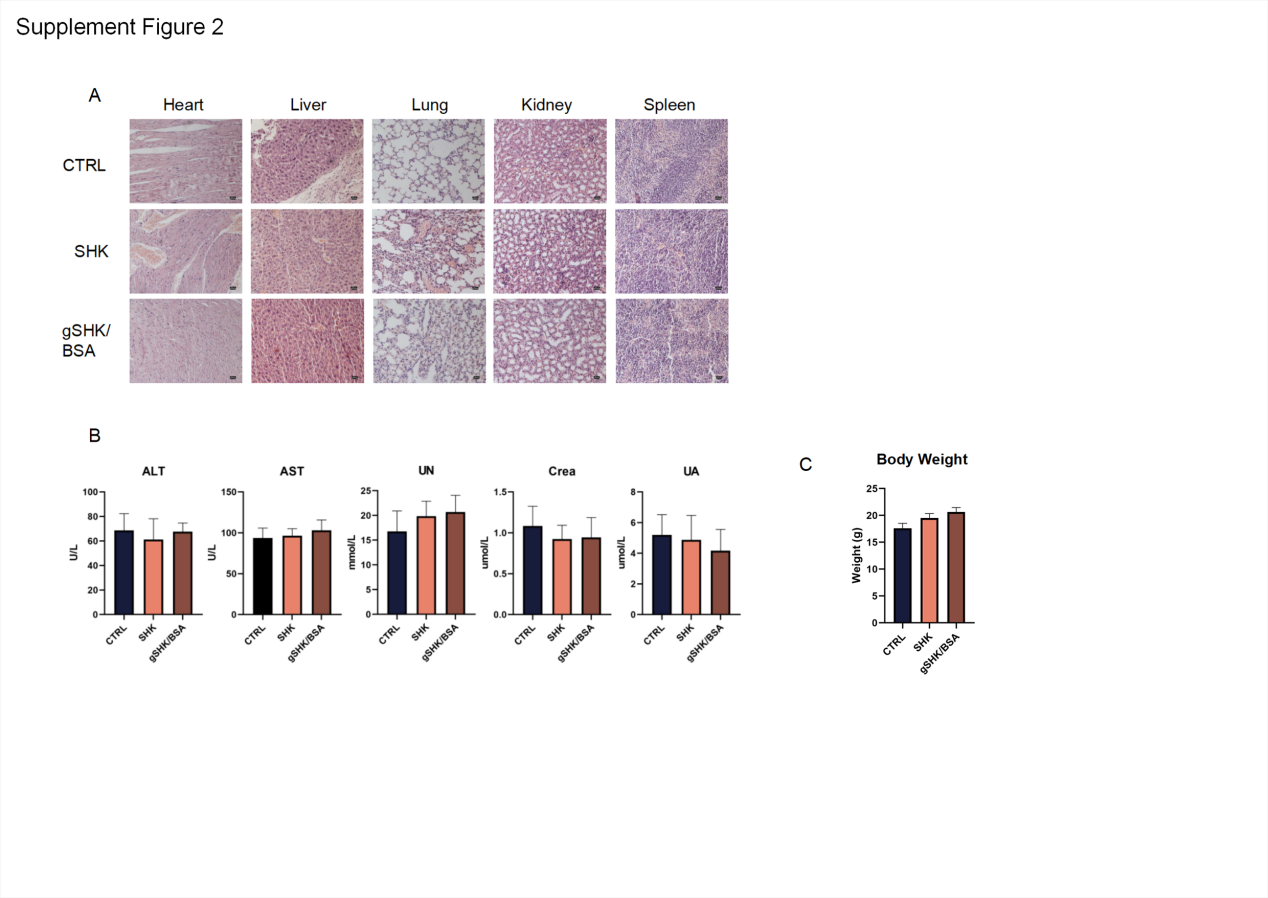
**

**Supplement Figure 2**

**A** H&E staining analysis of the heart, liver, lung, kidney, and spleen of mice receiving different drugs (CTRL, SHK, gSHK/BSA). **B** Blood biochemistry measurements were performed on lanine aminotransferase(ALT), aspartate aminotransferase(AST), urea(UN), creatinine(Crea), and uric acid(UA) in the blood of mice receiving different drugs (CTRL, SHK, gSHK/BSA). **C** Body weight of mice receiving different drugs (CTRL, SHK, gSHK/BSA) after 18 days.
